# Robust In Vitro Models for Studying Parkinson’s Disease? LUHMES Cells and SH-SH5Y Cells

**DOI:** 10.3390/ijms252313122

**Published:** 2024-12-06

**Authors:** Cameron Noah Keighron, Sahar Avazzedeh, Leo R. Quinlan

**Affiliations:** 1 Cellular Physiology Research Lab, School of Medicine, Department of Physiology, University of Galway, H91W5P7 Galway, Ireland; cameron.keighron@universityofgalway.ie (C.N.K.); sahar.ava@gmail.com (S.A.); 2 CÚRAM SFI Research Centre for Medical Devices, University of Galway, H91W2TY Galway, Ireland

**Keywords:** Parkinson’s disease, mitochondria, LUHMES, SH-SH5Y, electrophysiology, dopaminergic, neurodegeneration

## Abstract

As our population ages, there is an increased unmet clinical need surrounding neurodegenerative diseases such as Parkinson’s disease (PD). To tackle this ever-increasing problem, we must ensure that the cell models that we use to develop therapeutics in vitro are robust, reliable, and replicable. In this study, we compared SH-SY5Y cells with LUHMES cells in response to 6-Hydroxydopamine (6OHDA) and 1-Methyl-4-phenylpyridinium (MPP+), two common Parkinson’s insults used in in vitro analysis. Both these cell types have apparent dopaminergic phenotypes, which could aid us in understanding their potential in this race to novel therapies. The LUHMES cells showed consistent dopaminergic (DA) expression through tyrosine hydroxylase (TH) positivity, alongside depleted ATP levels and elevated reactive oxygen species (ROS) production, whereas the SH-SH5Y cells displayed resilience to both chemical insults, raising questions about their utility in accurately modelling PD pathology. Further electrophysiological analysis revealed comparable firing rates and ion channel signalling between both cell types; however, LUHMES cells demonstrated stronger calcium signalling responses, further supporting their use as a more robust PD model. While SH-SY5Y cells showed some adaptability in vitro, their inconsistent DA phenotype and limited response to chemical insults limit their suitability for advanced research, suggesting that LUHMES cells should and must take their place as a hallmark in Parkinson’s disease research.

## 1. Introduction

The hallmark of neurodegeneration is the gradual decline in brain function, often manifesting with overlapping biological and clinical symptoms [1]. Across the spectrum of neurodegenerative disease, there are many shared molecular pathways leading to common pathologies. The pathologies prominently feature protein aggregation, oxidative stress, neuroinflammation, and dysfunction of the blood–brain barrier [2,3,4]. These features contribute to the loss of neurons in various brain regions.

In the case of Parkinson’s disease (PD), dopaminergic (DA) neurons in the midbrain are especially vulnerable to degeneration [5,6]. PD is characterised by the loss of DA neurons and a deficiency in dopamine, which presents clinically as a general deceleration of normal movement (bradykinesia) and either a resting tremor or rigidity [7]. PD is categorised into early (stages 1 and 2) and late (stages 3 and 4). Stage 1 and 2 symptoms include rapid eye movement and sleep behaviour disorder (including sleep paralysis), with a reduction in the sense of smell, indicating initiation in the medulla and olfactory bulb [8,9]. As stages 3 and 4 progress, symptoms include cognitive impairment alongside challenges with movement and gait. At this stage, hallucinations are also present, suggesting the pathology has advanced to the substantia nigra pars compacta, as well as other midbrain and basal forebrain regions [10,11], often characterised by protein aggregations, including alpha-synuclein [12]. While some treatments for PD have been developed over recent decades, these are generally not disease-modifying and primarily support symptom management [13,14,15,16].

The pathobiological features of PD has many metabolic attributes that can be recapitulated in vitro and responses to potential therapeutics can be tested. These features include dysregulation in production and haemostasis of ATP, which is essential for energy supply, synaptic transmission, calcium homeostasis, axonal transportation, cellular repair, metabolic support, and neuroprotection [17,18,19,20]. Mitochondrial dysfunction is a major hallmark of not only PD but also neurodegeneration in general. It is one of the key pathologies researchers target in developing new therapeutic agents [21,22,23]. Reactive oxygen species (ROS) play a critical role in the pathogenesis of PD due to their contribution to mitochondrial dysfunction [24,25]. This leads to oxidative stress, which damages key mitochondrial components, impairing energy production. Understanding ROS levels in Parkinson’s models can reveal insights into the mechanisms of neurodegeneration, offering potential therapeutic targets for mitigating mitochondrial dysfunction and slowing disease progression [22,26,27].

There is an urgent need to develop novel and targeted therapeutics with the horizon of an ever-ageing population. The availability of physiologically relevant disease models is critical to developing new treatments. To date, SH-SH5Y cells have been a mainstay of pre-clinical in vitro modelling of PD in both 2D and 3D studies. The SH-SH5Y cell line was derived through three rounds of subcloning from the SK-N-SH neuroblastoma cell line, and the addition of specific compounds can induce their differentiation into various functional neuronal subtypes [28,29]. Retinoic acid, among other compounds, can drive SH-SH5Y cells into a dopaminergic phenotype; however, there is conflicting literature surrounding these cells’ true “dopaminergic” nature, even after differentiation [30,31]. Many researchers have relied on SH-SH5Y cells to study PD [28,29]. More recently, Lund human mesencephalic (LUHMES) cells have gained favour, as they represent human embryonic neuronal precursor cells capable of sustained proliferation, facilitated by the expression of a tetracycline-regulatable (Tet-off) v-myc transgene. LUHMES cells can be differentiated into mature dopaminergic neurons by adding tetracycline and dibutyryl cAMP [32,33].

In this study, we evaluate the effects of both 6-hydroxydopamine (6-OHDA) and 1-methyl-4-phenylpyridinium (MPP+) on LUHMES and SH-SH5Y cells in the context of neurodegeneration, in addition to assessing the electrophysiological profile of both cell types.

The 6-OHDA-induced Parkinson’s animal model is well established in the literature, with toxic effects observed peripherally and centrally [34]. Initially, the toxin accumulates within catecholaminergic neurons, which leads to a disruption of cellular homeostasis and neuronal damage. 6-OHDA is oxidised by monoamine oxidase (MAO-A), producing hydrogen peroxide (H_2_O_2_) [35,36]. H_2_O_2_ is cytotoxic but also initiates the production of oxygen-free radicals, leading to oxidative stress and, thus, cellular degeneration. MPP+ is another neurotoxin used in Parkinson’s research, known to induce mitochondrial dysfunction and apoptosis [37,38]. Like 6-OHDA, MPP+ is transported by the dopamine transporters and localises in the mitochondria, which leads to depleted ATP, reduced mitochondrial membrane potential, and, ultimately, cell death [37]. This paper aims to compare physiological responses in SH-SH5Y and LUHMES cells as “dopaminergic” models for understanding PD and generating new therapeutics.

## 2. Results

### 2.1. LUHMES Cells Are More Sensitive to PD-Associated Chemical Insults

**Figure 1:**
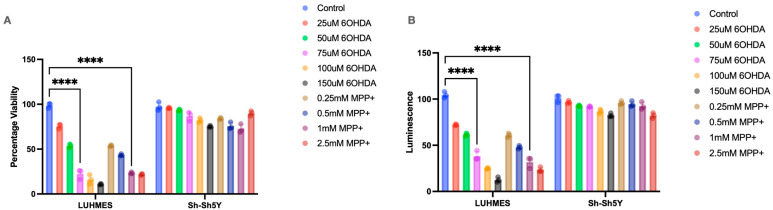
Effects of metabolic insults on cell viability and ATP production. (**A**) An Alamar blue assay measured viability, and significantly reduced viability was observed in differentiated LUHMES cells (n = 3). (**B**) ATP production was measured by CellTiter-Glo^®^ 2.0 luminescence assay, with a significant reduction in the ATP production in differentiated LUHMES cells (n = 3). Data are presented as means +/− sem, **** *p* < 0.0001).

**Figure 2:**
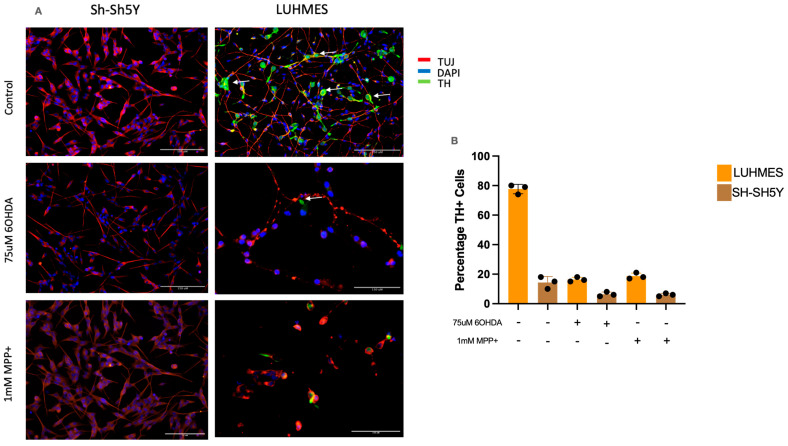
Differentiated LUHMES and SH-SH5Y cells show differences in tyrosine hydroxylase expression. (**A**) Representative images of SH-SH5Y and LUHMES cells treated with 75 μM 6-OHDA or 1 mM MPP+ (arrows point to areas of TH positivity, scale bar for all images is 150 μM). (**B**) Percentage of cells that are TH+ in treated and untreated SH-SH5Y and LUHMES cell cultures (n = 3).

The effects of 6OHDA and MPP+ were tested across a broad concentration range based on the extant literature [39,40,41,42]. LUHMES cells displayed significant sensitivity to 6-OHDA and MPP+ after 24 h, with a substantial reduction in viability detected (Figure 1A), alongside a change in morphology (Figure 2A). In contrast, SH-SH5Y cells were more resilient to the same concentrations and no significant change viability was detected; this was accompanied by a consistent, healthy morphology under the microscope (Figure 1A).

Through a luciferase assay, it was determined that the LUHMES cells’ ATP production was significantly impaired in response to both chemical insults (Figure 1B). However, the SH-SH5Y cells, again, were more resistant to these, demonstrated by the minimal reduction in ATP levels after 24 h of treatment (Figure 1B).

### 2.2. LUHMES Cells Are Positive for Tyrosine Hydroxylase

Dopaminergic neurons are tyrosine hydroxylase-positive (TH+) cells, where TH converts tyrosine into L-DOPA, the precursor to dopamine. These TH+ dopaminergic neurons are lost in neurodegenerative conditions such as PD. In this study, we show that post-mitotic LUHMES and SH-SH5Y cells were positive for the pan-neuronal maker TUJ in their outgrowths. Approximately 80% of LUHMES cells were also TH+ (Figure 2A), in contrast to SH-SH5Y cells, which had little if any TH expression (Figure 2A). Again in contrast to SH-SH5Y cells, the morphology of LUHMES cells was significantly altered post-treatment, with metabolic insults (Figure 2B). Several SH-SH5Y differentiation protocols were reviewed in the preparation of this work, with no significant difference detected (Table 1). For the remainder of this work, protocol 2, the most commonly reported protocol in the literature, was taken forward.

### 2.3. Metabolic Challenge Increases Reactive Oxygen Species (ROS)

**Figure 3:**
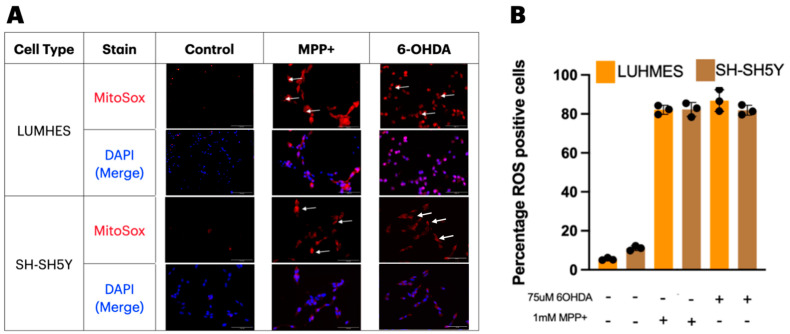
Mitochondrial superoxide production in response to metabolic insult. (**A**) Representative images of mitoSOX red and DAPI staining in 1 mM MPP+- and 75 μM 6-OHDA-treated cells (arrows point to areas of mitoSOX positivity, scale bar is 150 μM). (**B**) Percentage of mitoSOX red-positive cells for each treatment condition (n = 3). (Individual values are represented as black dots in graph **B**).

MitoSOX red selectively accumulates in the mitochondria and exhibits red fluorescence in live cells when oxidised by superoxide and was used here to visualise ROS change/production. The levels of ROS in SH-SH5Y and LUHMES cells was significantly increased when treated with 6-OHDA and MPP+ (Figure 3A). Image analysis revealed that 81% and 84% of LUHMES and SH-SH5Y cells, respectively, were MitoSOX positive after 24 h of 1 mM MPP+ treatment, with similar results reported for 6-OHDA treatment in both cell types (Figure 3B).

### 2.4. Population Electrophysiology Is Unaltered in Response to Metabolic Insults

**Figure 4:**
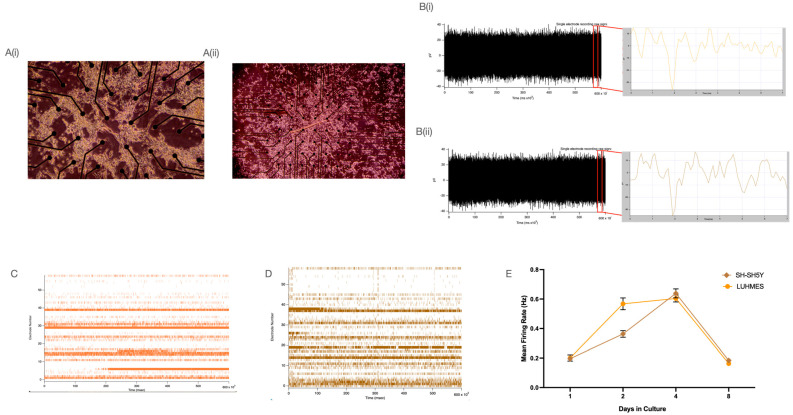
Multi-electrode array recording of SH-SH5Y and LUHMES cells. (**A**) Representative image of LUHMES cells (**i**) and SH-SH5Y cells (**ii**) on day 4 of differentiation (images taken at 20×). (**B**) Extracted spike data for LUHMES (**i**) and SH-SH5Y (**ii**) cells on day 4 differentiation. (**C**) Raster plot of the neuronal firing of LUHMES cells on day 4. (**D**) Raster plot of the neuronal firing of SH-SH5Y cells on Day 4. (**E**) Mean firing rate for SH-SH5Y and LUHMES cells (n = 3).

**Figure 5:**
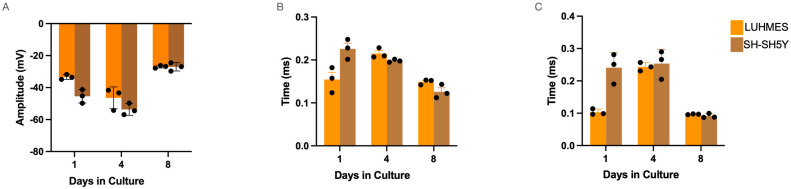
Multi-electrode array recording of LUHMES and SH-SH5Y cell continued. (**A**) Mean spike amplitudes across the differentiation timeline. (**B**) Calculated average rise time of each cell type across the differentiation timeline. (**C**) Calculated decay time of spikes across the differentiation timeline. (Individual values are represented as black dots in each graph).

Over eight days, differentiated neurons were monitored and the firing rate, spike height, average rise time, and decay time were extracted. The general morphology of the neurons after four days of differentiation on the glass MEAs was considered normal (Figure 4A). Cells were noted to lose adherence around day 8 and typically became fully detached by day 9 or 10. Isolated spike data were extracted on day 4 using a global threshold algorithm (crossing −35 pV) (Figure 4B). Multi-electrode array (MEA), a grid of electrodes that is used to record and stimulate electrical activity in neuronal cells, enables real-time analysis of cellular communication and electrophysiological patterns. MEA recordings were performed in 10 min epochs after 3 min of acclimatisation. Raster plots are shown below, noting all events over this threshold over time (Figure 4C,D). The mean firing rate (MFR) was calculated as the ratio of the total number of spikes recorded (n) and the duration of recording in seconds, *MFR* = *n*/*s*. The MFR for SH-SH5Y and LUHMES cells increased throughout the differentiation period and started to reduce as the neurons aged and detached from the glass coverslips (Figure 4E). No significant differences in the MFR were detected between cell types. This holds for all other parameters compared across both cell types. The average spike amplitude of the LUHMES and SH-SH5Y were −46.4 ± 6.8 (pV) and −53.7 ± 3.0 (pV), respectively (Figure 5A). On day 4, the average spike rise time for LUHMES and SH-SH5Y cells was 0.22 ± 0.01 ms and 0.20 ± 0.003 ms, respectively (Figure 5B). The spike decay time for LUHMES and SH-SH5Y cells was 0.24 ± 0.013 ms and 0.25 ± 0.04 ms (Figure 5C). Both cell types displayed similar profiles, peaking in spike activity at day 4 post differentiation, with a reduction in/loss of function by day 8.

### 2.5. Spontaneous and Activated Intracellular Calcium Dynamics

**Figure 6:**
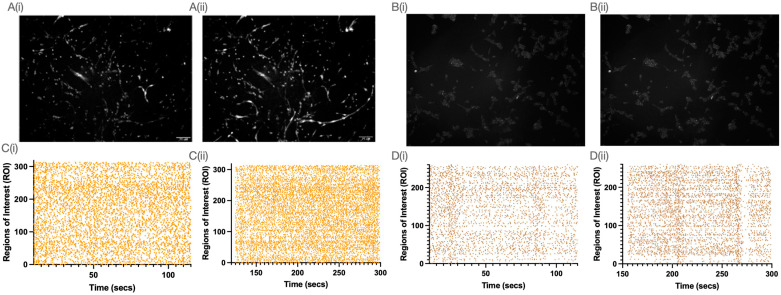
Calcium dynamics in SH-SH5Y and LUHMES cells. (**A**) LUHMES cells on day 4 of differentiation loaded with Fluo 4-AM, pre- (**i**) and post- (**ii**) ATP addition (images taken at 40×). (**B**) SH-SH5Y cells on day 4 of differentiation loaded with Fluo 4-AM, pre- (**i**) and post- (**ii**) ATP addition(images taken at 40×). (**C**) LUHMES cell raster plot of spikes over time pre- (**i**) and post- (**ii**) ATP addition. (**D**) SH-SH5Y cells raster plot of spikes over time pre- (**i**) and post- (**ii**) ATP addition.

**Figure 7:**
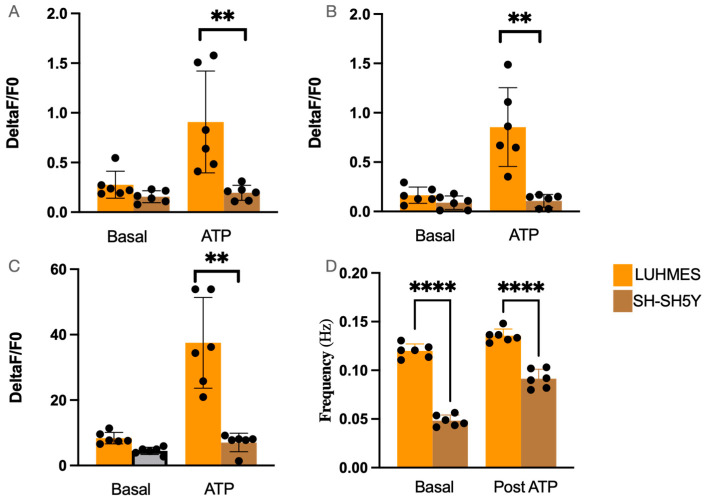
(**A**) Peak calcium response in LUHMES and SH-SH5Y cells, with both basal and ATP stimulated (n = 6). (**B**) Average calcium response in LUHMES and SH-SH5Y cells, with both basal and ATP stimulated (n = 6). (**C**) Area under the curve (AUC) of calcium response in LUHMES and SH-SH5Y cells, with both basal and ATP stimulated (n = 6). (**D**) Spike rate in LUHMES and SH-SH5Y cells, with both basal and ATP stimulated (n = 6). (Individual values are represented as black dots in each graph, Statistical significance was calculated using two-way ANOVA, ** *p* < 0.01 **** *p* < 0.0001).

Calcium signalling was recorded in SH-SH5Y and LUHMES cells, with analysis of their spontaneous response and response to ATP captured. Calcium signalling is central to neuronal function. In models of PD, including cells treated with toxins such as 6-OHDA or MPP+, mitochondria are often compromised. There was a significant difference in the uptake of Fluo 4-AM by either cell type (Figure 6A,B). LUHMES cells showed more basal calcium transients compared to SH-SH5Y cells, including in spikes detected over time (Figure 7A–D). Stimulating with exogenous ATP (0.1 mM) produced a robust response that was significantly larger in LUHMES cells (Figure 7A–D).

### 2.6. LUHMES and SH-SH5Y Cells Have Similar Inward and Outward Voltage Activated Currents

**Figure 8:**
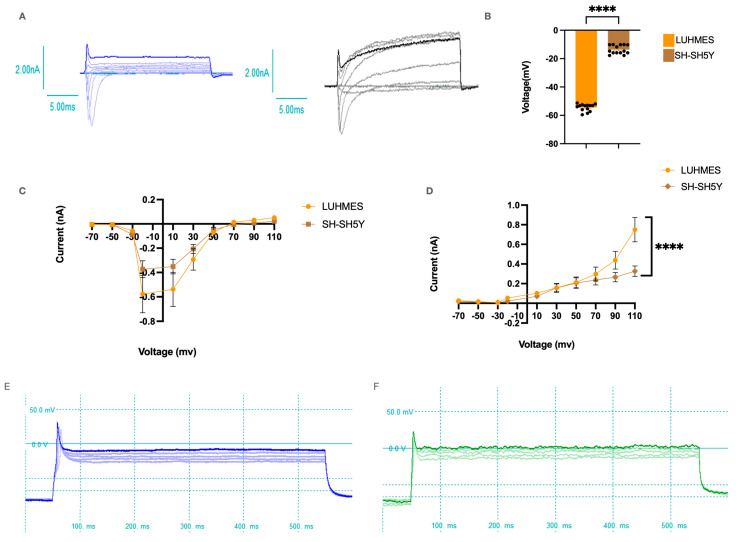
Single cell I/V relationships for SH-SH5Y and LUHMES Cells. (**A**) Representative current traces of the voltage clamp recording from LUHMES (n  =  8 cells, blue) and SH-SH5Y (n  =  8 cells, black), showing fast inward Na+ currents followed by slow outward K+ currents in response to different voltage steps applied. (**B**) Resting membrane potential of LUHMES and SH-SH5Y cells (n  =  14 cells). (**C**) Average outward current for both cell types. (**D**) Average inward current from both cell types. (**E**,**F**) Action potentials were recorded in both cell types, with E representing LUHMES and F representing SH-SH5Y cells. (Individual values are represented as black dots in graph B, Statistical significance was calculated using two-way ANOVA, **** *p* < 0.0001).

The automated Patchliner system (Nanion) was used to assess functional voltage gated channel activity. Under whole-cell recording conditions, there were significant differences in the resting membrane potential of both cell types (LUHMES:  −54.33 ±  0.003 mV; SH-SH5Y: −16.02  ±  0.009 mV), suggesting there are differences in their passive membrane properties. LUHMES and SH-SH5Y cells produced voltage-dependent current, which increased when voltage steps were applied (−70 mV to +110 mV, Figure 6A,B), with the Na+ current abolished under Na+-free conditions. There were no significant differences in the voltage-activated Na+ currents in either cell type. At 110mV, the outward K+ current was significantly higher in the LUHMES cells than in the SH-SH5Y cells (Figure 8C,D). Spontaneous APs were observed under current clamp mode with 0 pA current applied, with an average amplitude of 54.99 ± 3.29 (mV) for LUHMES cells and 60.484 ± 8.30 (mV) for SH-SH5Y cells (Figure 8B,E,F).

## 3. Discussion

The literature has widespread reports on the use of the human neuroblastoma cell line SH-SH5Y, which is probably the most commonly used in vitro model of dopaminergic neurons and seen as a basic first step in PD research [43]. Criticism of SH-SH5Y models is based on questioning how much of a “true” dopaminergic model they represent [28,44,45] when compared to more sophisticated models like iPSC-derived neurons. iPSC models have the advantage of allowing the pathology to develop rapidly and are especially useful in molecular and cellular studies. However, iPSCs present significant challenges, including genetic variability and difficult differentiation protocols that often result in immature neurons that can be labour-intensive and costly to generate. A more representative model than SH5-SH5Y and more simple and cheaper alternative to iPSCs would be ideal, and LUHMES cell may fill this gap. It is known that both 6-OHDA and MPP+ target mitochondrial dysfunction, leading to neuronal cell death [46,47,48], and it is essential in this context to compare both cell types’ metabolic viability and ATP production. Exploring this across a range of concentrations demonstrated a significantly higher sensitivity to both chemical insults for LUHMES cells, impacting their overall viability and ability to produce ATP after 24 h of exposure. However, the SH-SH5Y cells remained virtually unaffected by the treatments, including ATP levels that were relatively close to the untreated control. The SH-SH5Y cells’ higher resistance to chemical insults makes them a more complicated cell type to work with, as the increased concentration of the chemical insults, particularly 6-OHDA, has a detrimental effect on not only the mitochondria but also all other aspects of the cells [49,50,51,52]. The high level of tolerance makes it difficult to ascertain what is affected by mitochondrial dysfunction and what is affected by the increased cytotoxicity associated with increased concentrations of both chemical insults in SH5-SH5Y cells.

Beyond the metabolic profile, it was also essential to establish how “dopaminergic like” each cell type was through TH staining, an early and stable marker of DA neurons. This is in contrast to the dopamine transporter (DAT), which can be more variable in its expression [53,54]. To generate robust models for PD, it is preferable that cell models be consistently DA in nature. LUHMES cells showed consistent TH positivity from day 3 of differentiation, which was significantly impaired after adding chemical insults. Additionally, increased neuronal fragmentation was recorded in the LUHMES cells after exposure to the chemical insults. These results allow confidence in using LUHMES cells in PD research and confirm their reliable DA profile. On the contrary, SH-SH5Y cells displayed relatively low TH positivity and little to no fragmentation of the neurons after exposure to the same chemical insults. These results align with the metabolic results and place a question mark over the use of SH-SH5Y cells alone to study the development and potential therapeutics of Parkinson’s disease.

A core hallmark of neurodegeneration, including PD, is the increased production of reactive oxygen species (ROS) associated with mitochondrial dysfunction [55,56,57]. In this light, both cell types were evaluated for ROS using mitoSOX red. Both cell types showed a significant increase in ROS production after 24 h of exposure to 6-OHDA and MPP+. While the SH-SH5Y cells were resistant in terms of ATP production and viability, they may still be a good avenue to understanding ROS production. Here, LUHMES cells, consistent with previous results, showed an increase in ROS production in response to both chemical insults, indicating they may be a good tool for modelling PD.

In addition it is essential to understand how these neurons function physiologically [58,59]. Cell population (multi-electrode array) and single-cell (patch clamp) electophysiology and calcium dynamics are key measures of physiological phenotype. Some literature looks at some or all of these techniques in both cell types, but very little directly compares them alongside each other. Multi-electrode arrays enable simultaneous recording of electrical activity from multiple neurons. Both LUHMES and SH-SH5Y cells exhibited similar firing patterns over the eight-day differentiation protocol, which showed a sharp decline on day eight. However, this decline in firing potential is not entirely due to a loss of ability. It can also be attributed to cell loss as they detach and come away from the electrodes. Dopaminergic neurons had a mean firing rate (MFR) of between 0.1 and 1 Hz when analysed [60,61,62], with both cell types falling within this range, indicating they are both excellent MEA candidates in the study of PD and the development of new therapies. MEA analysis also offers a window into more than just how frequently these neurons are firing, including the average spike height of a firing event, the decay time of each spike, and the rise time of these firing events, with no significant difference detected from either cell type, suggesting that both cell types could be excellent candidates for studying the effect of PD and therapeutics on the electrical activity of neurons and network connectivity in real time across a population of neurons.

Functional voltage gated ion channel activity was also evaluated, as dysregulation of ion channels can disrupt neuronal excitability, impair neurotransmission, and lead to neuronal dysfunction or death [63,64]. Using an automated patch clamp, we assessed voltage-clamp responses in both cell types, with LUHMES and SH-SH5Y cells displaying similar ion channel profiles across days 6–8 of differentiation. Both cells possessed voltage-gated Na+ and K+ channels. There was only one point of significance detected in outward potassium current at the most depolarised potential tested (110 mV). These data suggest that both of these cell types could be used to study ion channel dysregulation. Calcium signalling plays a crucial role in Parkinson’s research, as it regulates neuronal communication, synaptic function, and mitochondrial health. Dysregulated calcium homeostasis is linked to neuronal death, particularly in dopaminergic neurons, which are highly sensitive to calcium overload [18,65,66]. We evaluated changes in intracellular calcium dynamics within both cell types, as this can help us identify and understand which cellular pathways are impaired [67,68,69]. LUHMES cells displayed spontaneous calcium signalling and a robust response to purinergeric stimulation (0.1 mM ATP). These data would strongly suggest that LUHMES cells are a better model cell for understanding the changes in calcium flux in PD and unlocking insight into the effectiveness of potential therapies.

Overall, both cell types show utility for studies to model Parkinson’s disease and understand the impact of potential therapeutics. However, consistent with the literature, SH-SH5Y cells are variable in their ability to provide a full and clear picture of the effects of neurodegeneration on dopaminergic neurons. Without a robust TH+ phenotype, it is hard to argue that SH-SH5Y cells are truly dopaminergic. In balance, LUHMES cells show versatility and adaptability in all techniques researchers need to use to evaluate the neurodegeneration associated with PD. Alongside this, they display a consistent, replicable dopaminergic phenotype. To this end, we recommend that LUHMES cells are the more robust, reliable cell type for developing novel therapies for and better understanding of PD.

## 4. Materials and Methods

### 4.1. Cell Culture

SH-SY5Y (SK-N-SH—ATCC HTB-11) human neuroblastoma cells were cultured in a growth medium of Dulbecco’s modified Eagle’s medium (DMEM) with 10% foetal bovine serum (FBS) and 1% antibiotic penicillin-streptomycin. Cells were seeded in T75 culture flasks (Sarstedt) for routine passaging in a 37 °C, 5% CO_2_, humidified incubator. The medium was replenished every three days. SH-SY5Y cells were differentiated by retinoic acid (RA) treatment. Briefly, cells were exposed to SH-SY5Y growth medium with reduced serum (1%) supplemented with 10 μM RA for five days.

LUHMES (obtained from Professor Marcel Leist, Leist Lab, Universitätsstraße 10, 78464 Konstanz, Germany) cells were plated on T-75 culture flasks pre-coated with 50 μg/mL poly-L-ornithine (PLO; Sigma-Aldrich, New Road, Gillingham, Dorset, SP8 4XT, UK) and 1 μg/mL fibronectin from human plasma (Sigma-Aldrich, New Road, Gillingham, Dorset, SP8 4XT, UK). Cells were maintained growth medium of advanced DMEM/F12 supplemented with 1% N–2 supplement (Gibco), L glutamine, and 1% penicillin-streptomycin in a 37 °C, 5% CO_2_, humidified incubator. The medium was replenished every three days. LUHMES cells were differentiated based on previously reported protocols. LUHMES cells were exposed to a differentiation medium consisting of advanced DMEM/F12, L-glutamine, 1 mM dibutyryl-cAMP (dbcAMP; (Sigma-Aldrich, New Road, Gillingham, Dorset, SP8 4XT, UK)) 1 μg/mL tetracycline hydrochloride (Sigma-Aldrich, New Road, Gillingham, Dorset, SP8 4XT, UK), and 1% penicillin-streptomycin for four days.

### 4.2. Cell Viability

Briefly, cells were seeded in 96-well plates at densities of 25 × 10^3^ (SH-SH5Y) and 40 × 10^3^ (LUHMES) after 24 h to allow for adhesion, and the medium was changed for the respective differentiation protocols. Once differentiation was complete, 6OHDA and MPP+ treatments were added for a treatment period of 24 h. After 24 h and to measure viability, both Sh-SH5Y and LUHMES culture medium was replaced with phosphate-buffered solution containing 10% (*v*/*v*) Alamar blue, and cultures were incubated for 3 h at 37 °C in humidified 95% air/5% CO_2_. The plates were visually inspected for a colour change from blue to pink in control wells. Alamar blue fluorescence was measured in a Hidex automated plate-reading fluorometer, with excitation at 530 nm and emission at 590 nm.

### 4.3. Cell Glo Titer 2.0 Reagents Assay for ATP Production

LUHMES and SH-SH5Y cells were seeded in a 96-well plate and incubated under standard differentiation culture conditions to allow for adherence and maturity. Cell Glo Titer 2.0 (Promega, Southhampton Science Park, Chilworth, S016 7QJ, UK) reagents were prepared by mixing luciferase, luciferin, and buffer components to produce a luminescent signal proportional to ATP levels. The mixed reagent (4 μL) was added to each well, and the plate was shaken briefly to ensure mixing. Luminescence was measured using a plate reader, with signal intensity directly correlating to cell viability. After this, the plate was read at 30 min, 6 h, 24 h, 48 h, and 72 h on a Hidex Plate reader.

### 4.4. Fluorescence Microscopy

To confirm the presence of DAergic characteristics, cells were seeded in 6 healthy plates (250 × 10^3^ cells per well) and their respective differentiation protocols were completed. Cells were treated with 1 mM MPP+ and 75 μM 6-OHDA alongside untreated controls. Twenty-four hours after treatment with chemical insults, SH-SY5Y and LUHMES cells were fixed in 4% paraformaldehyde for 20 min. The cells were then permeabilised and blocked with 0.2% Triton X-100 and 0.1% bovine serum albumin in PBS for 1 h. The cells were then incubated in anti-beta tubulin (1:1000) and tyrosine hydroxylase (1:1000) at 4 degrees overnight. The cells were then incubated with Alexa 488 anti-mouse and Alexa 555 anti-rabbit secondary antibodies for 1 h at room temperature. After this, cells were incubated for 15 min in DAPI (1:1000) to label the nucleus of each cell. Labelled cells were imaged using EVOS microscopy equipment (M7000, Version 2) (Thermo Fisher, Bishop Meadow Road, Loughborough, LE11 5RE, UK) software. Images were then analysed using ImageJ software (Version 2.9.0).

### 4.5. Superoxide Production Assay

MitoSOX Red (Invitrogen, Waltham, Massachusetts, U.S, 5 μM in DMSO) was used to detect mitochondrial superoxide. Cells were seeded in 12-well plates on glass coverslips at 125 × 10^3^ cells per well. Twenty-four hours after adhesion, both cell types underwent specific differentiation protocols. Cells were treated with 1 mM MPP+ and 75 μM 6-OHDA. Twenty-four hours after treatment, media were removed and MitoSOX Red (5 μM) diluted in the respective culture medium were added for 15 min. After this, cells were incubated for 15 min with DAPI (1:1000 in culture medium) to label the nucleus of each cell. Cells were immediately imaged at 20× on EVOS microscopy equipment and software. Images were then analysed using ImageJ software (Version 2.9.0).

### 4.6. Multi-Electrode Array (MEA) Recordings

Microelectrode arrays (60MEA200/30iR/T, Multi-Channel Systems MCS GmbH, 72770 Reutlingen, Germany) recorded neuronal activity. Briefly, microelectrode arrays were coated with 1 mL 50 μg/mL poly-L-ornithine (PLO; Sigma-Aldrich) and 1 μg/mL fibronectin from human plasma (Sigma-Aldrich) for 24 h at 37 °C. After removing the coating solution, arrays were air-dried for 10 min in the cell culture hood before 1 × 10^6^ SH-SH5Y or LUHMES cells were added in 20 μL droplets to the centre of each MEA and placed in the cell culture incubator for 1 h. Arrays were then flooded with 1 mL of differentiation medium. Cells were differentiated for 5 days before measuring neuronal activity across multiple days. MEA recordings were analysed using the Neuromatic plugin [70] on Igor Pro 8 (Wavemetrics, Version 8).

### 4.7. Calcium Imaging

Cells were seeded on glass coverslips in 12-well plates. After 24 h, media were changed to differentiation media. Cells were then treated with various concentrations of chemical insults for 24 h. After this, the medium was removed, and cells were washed with artificial cerebrospinal fluid (ACSF) and replaced with Fluo-4-AM diluted in DMEM for 20 min. Cells were washed with ACSF and incubated in DMEM for 15 min before imaging. Glass coverslips were mounted onto the calcium chamber, flooded with ACSF, and placed on the microscope. Occular software (version 2.0) controlled the camera, and 5 min recordings were taken, including 2 min of baseline, and stimulated with 0.1mM ATP. These recordings were then analysed using Sarfia and Igor Pro 8 (Wavemetrics).

### 4.8. Automated Patch Clamp Recordings

All recordings were conducted in the whole-cell configuration using the Nanion*. The intracellular solution used consisted of 10 mM EGTA (CAS.No.67-42-5 purity: 99%), 10 mM Hepes (CAS.No. 7365-45-9 Purity: 99.5%), 10 mM KCl (CAS.No. 7447-40-7 purity: 99.5%), 10 mM NaCl (CAS.No. 7647-14-5 purity: 99.8%), and 110 mM KF (CAS.No. 7789-23-3 purity: 99%) adjusted to pH 7.2 with KOH (CAS.No. 1310-58-3 mOsm > 280). The extracellular solution used consisted of 140 mM NaCl (CAS.No. 7647-14-5 purity: 99.8%), four mM KCl (CAS.No. 7447-40-7 purity: 99.5%), 5 mM D-Glucose monohydrate (CAS.No. 14431-43-7 purity: 99.5%), and 10 mM Hepes (CAS.No. 7365-45-9 purity: 99.5%) adjusted to pH 7.4 with NaOH (CAS.No. 1310-73-2 mOsm 289). Medium-resistance, one-hole-per-well chips were tested to determine the optimal recording condition and to achieve whole-cell configuration. Voltage protocols were constructed using PatchControl 384 (Nanion Technologies, Livingston, NJ, USA). Initially, currents were evoked using a voltage step protocol from the holding potential of –80 mV with 10 sweeps starting at −100 mV to +80 mV. Cells were then hyperpolarised at −120 mV for 20 ms before currents were evoked using another voltage step protocol with ten sweeps starting at −70 mV to +110 mV. Lastly, action potentials were assessed using a current clamp protocol with an injection of 5 rounds of different currents starting at +20 amps to +100 amps. Data were analysed, and points were plotted as mean peak amplitude ± SEM for current-voltage plots.

### 4.9. Statistical Analysis

Statistical analysis was performed using GraphPad Prism (version 6, GraphPad Software, Inc., San Diego, CA, USA). Each experiment was performed in triplicate, and values are expressed as the mean ± standard deviation (SD) or ± SEM. Statistical significance is denoted as **** for *p* < 0.0001.

## 5. Conclusions

The number of people globally who will be diagnosed with PD or other neurodegenerative diseases is set to increase over the next ten years. The models used to study this disease must be evaluated to ensure they still hold relevance. SH-SH5Y cells have been a hallmark of PD research for the last number of years. However, looking at the data presented in this paper, it is perhaps time for this model to be updated in favour of LUHMES cells. While both cell types share some similarities in their biological and electrophysiological profiles, their differences set them apart. The LUHMES cells have consistent and reliable dopaminergic expression, are more sensitive to chemical insults, and offer us a critical window into the calcium dynamics of PD. Overall, LUHMES cells give us a well-rounded opportunity to generate accurate physiologically relevant models of PD.

## Figures and Tables

**Table 1:** Overview of results from three separate SH-SH5Y differentiation protocols. No significant differences were detected between any protocol trialled. These protocols were selected based on previous work noted in the literature. All experiments include n = 3 replicates.

	Protocol 1 (10 μM Retinoic Acid Only)	Protocol 2 (10 μM Retinoic Acid and Reduced Fetal Bovine Serum)	Protocol 3 (10 μM Retinoic Acid, Reduced Fetal Bovine Serum, and BDNF)
Reduced Metabolic Viability	ns	ns	ns
Reduced ATP Production	ns	ns	ns
Tyrosine Hydroxylase Production	Negative	Negative	Negative
Reactive Oxygen Species (Mitosox)	Positive	Positive	Positive

## Data Availability

The original contributions presented in this study are included in the article. Further inquiries can be directed to the corresponding author.
